# Recognition of extremophilic archaeal viruses by eukaryotic cells: a promising nanoplatform from the third domain of life

**DOI:** 10.1038/srep37966

**Published:** 2016-11-28

**Authors:** Kristine Buch Uldahl, Linping Wu, Arnaldur Hall, Pavlos Papathanasiou, Xu Peng, Seyed Moein Moghimi

**Affiliations:** 1 Danish Archaea Centre, Department of Biology, University of Copenhagen, Ole Maaløes vej 5, Copenhagen, 2200, Denmark; 2 Nanomedicine Research Group, Centre for Pharmaceutical Nanotechnology and Nanotoxicology, Department of Pharmacy, University of Copenhagen, Copenhagen, Denmark; 3 School of Medicine, Pharmacy and Health, Durham University, Wolfson building, Queens campus, Stockton on Tees, TS17 6BS, UK

## Abstract

Viruses from the third domain of life, *Archaea*, exhibit unusual features including extreme stability that allow their survival in harsh environments. In addition, these species have never been reported to integrate into human or any other eukaryotic genomes, and could thus serve for exploration of novel medical nanoplatforms. Here, we selected two archaeal viruses *Sulfolobus* monocaudavirus 1 (SMV1) and *Sulfolobus* spindle shaped virus 2 (SSV2) owing to their unique spindle shape, hyperthermostable and acid-resistant nature and studied their interaction with mammalian cells. Accordingly, we followed viral uptake, intracellular trafficking and cell viability in human endothelial cells of brain (hCMEC/D3 cells) and umbilical vein (HUVEC) origin. Whereas SMV1 is efficiently internalized into both types of human cells, SSV2 differentiates between HUVECs and hCMEC/D3 cells, thus opening a path for selective cell targeting. On internalization, both viruses localize to the lysosomal compartments. Neither SMV1, nor SSV2 induced any detrimental effect on cell morphology, plasma membrane and mitochondrial functionality. This is the first study demonstrating recognition of archaeal viruses by eukaryotic cells which provides good basis for future exploration of archaeal viruses in bioengineering and development of multifunctional vectors.

Viruses are receiving increasing attention as novel nanoplatforms with applications in materials science and medicine^1^. Viruses demonstrate remarkable features including plasticity, coordinated assembly, and site-specific delivery of nucleic acids. Viruses are also amenable to genetic engineering, their internal cavity may be filled with therapeutic agents, and the functional groups on the virus capsid may be modified with biomolecules, synthetic polymers and diagnostic agents^2^. Accordingly, viruses could provide basis for the development of alternative multifunctional vectors and theranostic platforms^1^,^3^. Within such notions, plant viruses and bacteriophages receive special attention, as they are considered non-infectious and non-hazardous in humans^4^. Another group of viruses that fits this criterion is archaeal viruses, a highly diverse and abundant category of viruses from the third domain of life, Archaea^5^, yet their potential remains untapped.

Archaeal viruses offer an ideal search pool for novel nanoplatforms as they have several attractive features. They are non-pathogenic, offer unique morphologies, and have specializations to survive in extreme environments^6^. All known archaeal viruses infect extremophilic archaea, and are thus adapted to survive the harsh environments of the host, making them extremely stable entities^7^,^8^. As a group, archaeal viruses show distinct morphologies not found in bacteriophages or plant viruses. These include spindle-, bottle-, and droplet-shape^6^. Accordingly, due to their unique shapes and inherent properties, archaeal viruses may prove as interesting vehicles for differential targeting of eukaryotic cells. Furthermore, size and shape have been identified as key factors influencing circulation half-life, biodistribution and cellular uptake of particulate drug delivery vehicles^9^,^10^. Although several articles suggest archaeal viruses as promising nanoplatforms^7^,^11^,^12^, to the best of our knowledge no studies have investigated the uptake and intracellular fate of any archaeal virus by human cells; a first step in evaluating their potential as a nanoplatform for cellular targeting and manipulation.

Here, we studied two archaeal viruses; *Sulfolobus* monocaudavirus 1 (SMV1) and *Sulfolobus* spindle shaped virus 2 (SSV2) as candidate nanoplatforms. Both viruses infect hosts from the archaeal genus *Sulfolobus* which are found in volcanic hot springs, and are considered hyperthermophilic acidophiles with optimal growth at 80 °C and pH 2–3^13^. The fusellovirus SSV2 is a dsDNA virus with a genome size of 14.8 kb^14^. The virion is spindle-shaped. This shape is only found among archaeal viruses. The virion body has short flexous fibers at one pole and is ~60 nm in diameter. SMV1 shares morphological similarity with SSV2, but it is significantly larger (120 nm) with a genome size of 48.8 kb^15^,^16^. SMV1 and SSV2 were selected owing to their unique spindle-shape, hyperthermostable and acid-resistant nature. Furthermore, both species are well-established laboratory strains with the potential for up scaling. We have investigated the uptake, intracellular fate, and safety of fluorescently labelled SMV1 and SSV2 in two different endothelial cell types of human origin: hCMEC/D3 and HUVEC, providing the first insights into the interaction between archaeal viruses and eukaryotic cells.

## Materials and Methods

### Production and purification of virus particles

SSV2 was propagated in *S. solfataricus* 5E6, a host for different viruses as described previously^17^. SMV1 was propagated in *S. islandicus* ΔC1C2^18^. Both host cultures were grown in *Sulfolobus* medium supplemented with 0.2% (w/v) tryptone, 0.1% (w/v) yeast extract, 0.2% (w/v) sucrose and 0.002% (w/v) uracil (TYS + U medium)^13^. Cultures were started from −80 °C stock; cells were transferred to 50 mL TYS + U medium and incubated at 78 °C. After 24 h of propagation, the cell culture was transferred to 950 mL of pre-heated (78 °C) TYS + U medium. The culture was grown to an OD_600_ of 0.2–0.3 (typically 24 h) at which time-point the host culture was infected with virus isolate (MOI 0.1). The supernatants containing the virus particles were collected 48–72 h post-infection and concentrated by ultrafiltration using 1,000 kDa molecular-weight cut-off (MWCO) centrifugal filter units (Sartorius, Aubagne Cedex, France). Additionally, the virus particles were purified by ultracentrifugation through a 10–40% (w/v) continuous Iodixanol gradient. Continuous gradients were prepared by sequentially layering 10, 20, 30 and 40% (w/v) Iodixanol solution (OptiPrep™, Axis-Shield PoC AS, Oslo, Norway) in 10 mM Tris-HCl, pH 6.0 into 14 mL centrifuge tubes (Beckman Coulter UK Ltd., High Whickham, Bucks, UK). The gradients were left in the dark at 4 °C overnight and then concentrated virus preparations were layered over the gradient (1.0 mL) and centrifuged in a SW-41 rotor (Beckman Coulter) for 6 h at 95,000 *g* at 4 °C. An opaque band was visible and recovered and an additional concentration step was performed. The virus fraction was washed three times with 10 mL of 10 mM Tris-HCl, pH 6.0 to remove Iodixanol and to prevent interference with downstream processes. Transmission electron microscopy (TEM) was used to confirm the presence of viral particles in the recovered fractions, and the number of plaque forming units (PFU)/mL was calculated (see below). The virus preparations were then stored at 4 °C until used.

### Virus particle labelling

For labelling of viruses, 1.0 μL of PKH67 (Sigma, St. Louis, MO), a fluorescent membrane dye that efficiently labels enveloped viruses non-covalently, was dissolved in 1.0 mL of Diluent C (Sigma, St. Louis, MO) immediately before the start of procedures^19^. Two volumes of diluted PKH67 was added to one volume of viral suspension (250.0 μL), and mixed by pipetting. After 3 min, the reaction was stopped by adding three volumes of 1% (w/v) BSA, then incubated for 1 min to allow binding of excess dye. The suspension was then centrifuged at 20,000 *g* for 20 min at 4 °C and the supernatant were discarded. Labelled virus were re-suspended in 3 mL of 10 mM Tris-HCl, pH 6.0 and centrifuged at 20,000 *g* for 20 min. The washing step was repeated and the final pellet were re-suspended in 250.0 μL Tris-HCl, pH 6 and stored at 4 °C in the dark.

### Determination of virus size and concentration

NanoSight LM20 Nanoparticle Tracking Analysis (NTA) (NanoSight, Amesbury, UK), equipped with a sample chamber with a 405 nm blue laser and a Viton fluoroelastomer O-ring, was used for determination of the hydrodynamic size distribution and viral concentration before and after labelling^20^. All measurements were performed in triplicate and at room temperature.

### Transmission electron microscopy

Viral preparations were placed on a carbon-coated copper grid for 5 min, and stained with 2.0% (w/v) uranyl acetate for 30 s. Images were recorded using a JEM-1010 transmission electron microscope (JEOL, Tokyo, Japan) with a Gatan digital camera 792.

### Plaque assays

The approximate virus titer before and after fluorescent labelling was determined by plaque assays. Serial dilutions (10.0 μL) of viral preparations were mixed with a sample (2.0 mL) of the exponentially growing host culture (2 × 10^8^ cells); *S. islandicus* ΔC1C2 for SMV1 infection and *S. solfataricus* 5E6 for SSV2 infection, respectively. The mixture was incubated for 30 min at 78 °C to allow the adsorption of the virus to the host cells. Immediately following the addition of 2 mL of TYS + U medium containing 0.25% (w/v) Gelrite (78 °C), the sample was layered over a premade 0.8% (w/v) Gelrite plate (78 °C). The plates were incubated for 2 days at 78 °C. SMV1 plaques appeared as small clear halos, whereas SSV2 plaques appeared turbid making them difficult to detect and enumerate.

### Imaging of infected host cells

From an exponentially growing *S. islandicus* ΔC1C2 culture (OD_600_ ~ 0.2), 5.0 mL were collected and incubated with SMV1 purified stock at an MOI of 10. At 5 and 30 min post infection (mpi) 1.0 mL cells were pelleted at 8,000 *g* for 5 min and then re-suspended in 500 μL 4% (v/v) formaldehyde. After 10 min incubation at room temperature, cells were pelleted at 8,000 *g* for 3 min then washed with PBS buffer, pH 7.4. To permeabilize cells, pellets were suspended in 500 μL PBS containing 0.1% (v/v) Triton X-100 and incubated at room temperature for 15 min. Cells were then washed three times with PBS to thoroughly remove all traces of the detergent. Pellets were re-suspended in 100–200 μL PBS, and 10.0 μL were spread onto poly-D-lysine coated coverslips. After air-drying, the coverslips were washed with PBS then stained with DAPI (10.0 μM) for 15 min at room temperature. After staining cells were washed three times with PBS, a cover glass was placed on top and the edges were sealed with clear nail polish. Coverslips were stored in the dark at 4 °C. Differential interference contrast and fluorescent images were acquired in Volocity (Improvision) with a cooled Orca-ER CCD camera (Hamamatsu) mounted on a Zeiss AxioImager Z1 microscope (Carl Zeiss, Germany). All images were captured at 100-fold magnification using a Plan-Apochromat 100×, 1.4 NA objective lens.

### Cell uptake studies

Human brain capillary endothelial cell line hCMEC/D3, transduced by lentiviral vectors incorporating human telomerase or SV40 T antigen, were grown in EBM-2 medium supplemented with 5.0% (v/v) FBS, hydrocortisone (1.4 × 10^−6^ M), basic fibroblast growth factor (1 ng/mL), 1.0% (w/v) penstrep and 10 × 10^−9^ M HEPES^16^. Human umbilical vein endothelial cells (HUVECs) were grown in DMEM with 20.0% (v/v) FBS on gelatin-coated flasks. All cells were maintained at 37 °C in a humidified atmosphere (air supplemented with 5% CO_2_). For the uptake study, hCMEC/D3 cells and HUVEC cells (2 × 10^4^/cm^2^) were seeded on 24-well plates (Corning, USA) and grown for 24 h at 37 °C, and 5% CO_2_ to 60–70% confluence. Afterwards, cells were washed three times with pre-warmed PBS and the uptake studies were initiated by adding different concentrations of PKH67 labelled viral particles (2.9–23.5 × 10^10^ particles/mL). After viral challenge for 24 hours, the medium was removed and cells were washed three times with pre-warmed PBS and harvested by trypsinization. A total of 10,000 cells, suspended in ice-cold PBS, were analyzed by flow cytometry (FACS Array Cell Analysis, BD, USA). For live-cell microscopy and organelle tracking, cells were seeded on eight-well Lab-Tek chamber slides (Nunc, Naperville, IL) for 1 day and then labelled with CellLight Reagents BacMam 2.0 Early endosomes-GFP, Lysosomes-GFP, Golgi-GFP, Endoplasmic reticulum (ER)-GFP, and Tubulin-GFP in accordance with the manufacturer’s protocol. Live cell imaging was performed on a Leica AF6000LX microscope equipped with a 63× (numerical aperture 1.47) oil objective using 1.6× magnification and analysed^20^. The co-localization analysis was processed with Image J to calculate Manders’ coefficient as described previously^20^.

### Cell functionality and viability tests

Lactate dehydrogenase (LDH) release, and cell respiration was investigated as described in detail elsewhere^20^,^21^. LDH release from hCMEC/D3 cells and HUVECs was measured at 24 h post incubation with virus using CytoTox96 Non-Radioactive Cytotoxicity Assay kit (Promega). The possible effects of viruses on cellular respiration were investigated by using XFe96 extracellular flux analyzer and XF96 V3 cell culture microplates (Seahorse Bioscience)^21^. Briefly, cells were seeded at 1.0 × 10^4^ cells/well in growth medium for 24 h. and thereafter challenged with viruses at 37 °C and 5% CO_2_ for 24 h. The following day, oxygen consumption rate (OCR) was measured at 37 °C in Seahorse assay buffer (containing 10 mM glucose, 10 mM pyruvate, at pH 7.4). Different mitochondrial respiratory states were examined through sequential addition of different compounds and corrected for non-mitochondrial respiration. To obtain estimations of mitochondrial quality following viral challenge, the respiratory ratio (RCR) and the coupling efficiency of OXPHOS were calculated as described elsewhere^21^,^22^. Mitochondrial inhibitors used to obtain mitochondrial respiratory states were applied at the following concentrations: oligomycin A (1 μM), carbonyl cyanide-4-(trifluoromethoxy)phenylhydrazone (FCCP; 1.0 μM), antimycin-A (2.5 μM) and rotenone (2.5 μM).

## Results and Discussion

### Viral morphology and size distribution after PKH67 labelling

**Figure 1:**
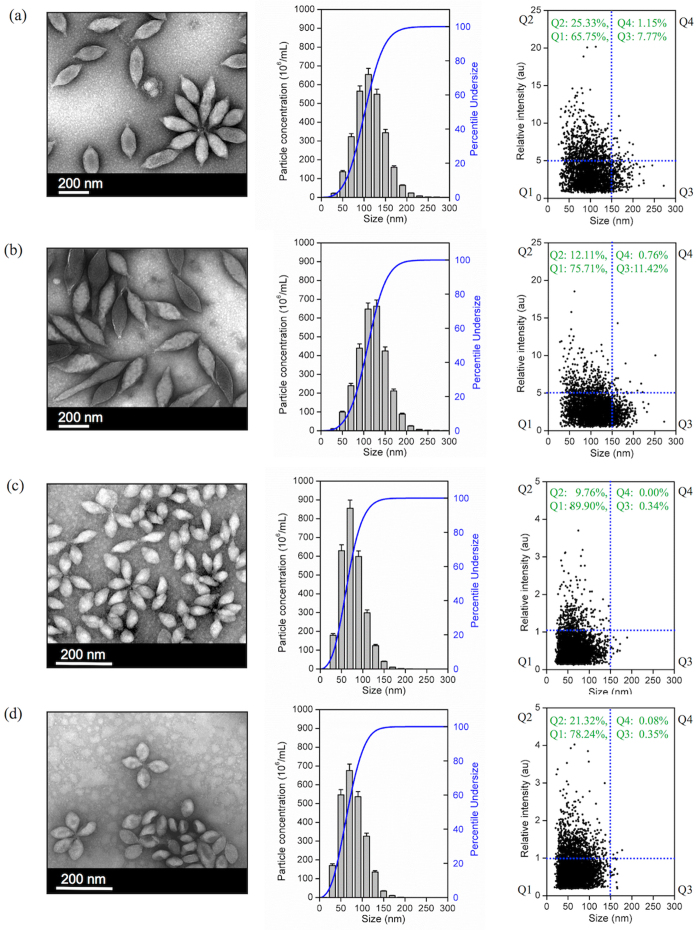
Physical characteristics of viruses before and after labeling with PKH67. (**a**) SMV1, (**b**) SMV1-PKH67, (**c**) SSV2, (**d**) SSV2-PKH67. Left panel: Transmission electron micrographs of viruses; Middle: Typical size distribution profile and concentration of viral preparations determined by Nanoparticle Tracking Analysis; Right panel: 2D plots of relative light scattering intensity of viruses versus the estimate of their size.

Morphological characteristics of SMV1 and SSV2 were studied by TEM and representative micrographs are presented in Fig. 1. SMV1 is spindle-shaped with an extending tail at one pole. SMV1 appears both individually and in a rosette-like arrangement, where virus-virus interaction appears to be mediated through the tail regions. Although, the general morphology of SMV1 remains unaltered after PKH67 labelling, some virions appear more electron dense. SSV2 is also spindle-shaped bearing flexous fibres at one pole, but they are smaller than SMV1 (Fig. 1). This species also form rosette-like structures of 3–6 virions as seen in TEM (Fig. 1). Again, their morphological characteristics appear to be preserved on PKH67 labelling and with some increase in electron density.

NanoSight LM20 Nanoparticle Tracking Analysis (NTA) was used for sizing and counting of viral preparations. NTA deduces the size on a particle-by-particle basis, relating the changing position of scattering viruses under Brownian motion^23^. NTA, however, assumes sphere equivalent hydrodynamic diameter particle size. Accordingly, SMV1 displays a narrow size distribution with a mean sphere equivalent hydrodynamic diameter of 114 ± 2 nm (n = 3) and 120 ± 1 nm (n = 3), with corresponding modes of 108 ± 2 nm and 120 ± 3 nm, before and after labelling, respectively (Fig. 1). NTA analysis further reveals existence of a small population of ≤50 nm particles, presumably arising from particulate contaminants and debris that could not be removed during the purification steps. This population, however, seems unaffected after labelling. Another interesting feature of the NTA analysis is the slight increase in the population of ≥150 nm particles after PKH67 labeling, which most likely is a reflection of aggregate formation among particles in the 75–100 nm ranges, since their concentration drops compared with untreated viruses. These differences are further reflected in scatter plots of relative intensity versus particle size, which allow differences in particle composition to be explored. The results reveal broad scattering of particles in 50–150 nm size ranges with relative intensities of 1–10 arbitrary units (au), whereas larger particles/aggregates predominantly scatter with relative intensities <5 au (Fig. 1). PKH67-labeling causes considerable drop in the proportion of high scattering (>5 au) SMV1 particles, and concomitantly increase the proportion of particles that scatter <5 au, including particles ≥150 nm in size.

NTA determination also shows a narrow size distribution for SSV2 with a mean sphere equivalent hydrodynamic diameter of 76 ± 1 nm (n = 3), and 75 ± 2 nm (n = 3), with corresponding modes of 63 ± 5 nm, and 65 ± 4 nm, before and after PKH67 labelling, respectively. Again, a small proportion of small particles (20–30 nm) are observable before and after labelling, which may represent the presence of some impurities (Fig. 1). Unlike SMV1 preparation, PKH67 did not increase the proportion of larger particles (>100 nm). Considering the hydrodynamic diameter of SSV2, the detection of a minor population of ≥150 nm may represent SSV2 rosettes that were observed in the electron micrographs. Finally, the scatter-plots show that the bulk of SSV2 particles scatter with intensities ≤1 au, but after labelling a higher proportion of SSV2 scatter >1 au (Fig. 1).

### Viral infectivity after PKH67 labelling

**Figure 2:**
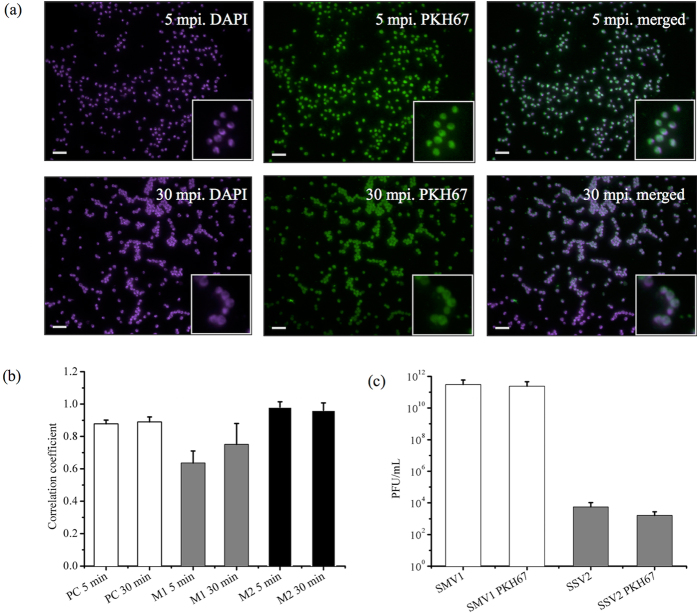
Infectivity of PKH67 labeled viruses. **(a)** Host cell imaging of *S. islandicus* ΔC1C2 infected with SMV1. Cells were stained with DAPI and SMV1 particles were labeled with PKH67. The co-localization of DAPI stained cells (blue, left panel) with virus particles (green, middle panel) appears as light purple (right panel). Bar = 9 μm. The insets in (**a**) represent magnified (2x) views of the selected regions. The Pearson’s and Mander’s overlap coefficients are represented as the average of 6 individual images of infected cells at initial stage of virus infection, 5 min post infection (mpi) and 30 mpi, at an MOI of 10 (Panel **b**). The infectivity was measured by a plaque assay before and after labelling, and presented as PFU/mL (Panel **c**).

We infected a culture of the archaeal host, *Sulfolobus islandicus* ΔC1C2, with PKH67 labelled SMV1 to investigate whether PKH67 signal colocalize with the host cells stained with DAPI. Co-localization analysis revealed a Pearson’s correlation coefficient (PCC) of ~0.9. Co-localization coefficients *M*_*1*_ and *M*_*2*_ demonstrated that ~95% of the green pixels (deriving from PKH67) co-localize with the blue DAPI pixels, whereas 65–75% of the blue pixels (host cells) co-localize with the green pixels (Fig. 2a,b). These observations suggest that at least the majority of the PKH67 signal is derived from the labelled SMV1, which remains infectious. Similarly, PKH67 labelled SSV2 exhibited colocalization with its host *S. solfataricus* 5E6 (Supplementary Fig. S1). In addition, we used *S. acidocaldarius*, a *Sulfolobus* species which is not a host to any known archaeal viruses, to confirm selective infectivity. Indeed, no co-localizing signals were observed with DAPI stained *S. acidocaldarius* cells which were incubated with PKH67-labeled SMV1 under the same condition as used for *S. islandicus* ΔC1C2 (Supplementary Fig. S2).

Potential interference of PKH67 labelling to the viral infectivity was examined by plaque assay with archaeal host cells, *S. islandicus* ΔC1C2 for SMV1 and *S. solfataricus* 5E6 for SSV2. The results in Fig. 2c confirm that both viruses remain infectious after PKH67 labelling, although SSV2 infectivity dropped about 4 folds after PKH67 labelling. This drop of infectivity after labeling might be due to the smaller size of SSV2 which makes it more difficult to pellet SSV2 than SMV1 under the same centrifugation condition. Moreover, the morphology of the plaques remained unchanged after PKH67 labelling (Supplementary Fig. S3). However, the plaquing efficiency of SSV2 is extremely low and virus titre measured by plaque assays is several orders of magnitude lower than actual viral counts, as observed in our laboratory and as reported previously by others^14^. On the other hand, SSV2 plaques appear unusually large and each plaque seems to be originated from multiple viral particles. Accordingly, the virus titre of SSV2 must be viewed cautiously and we used NTA counts instead for the following experiments.

Collectively, these observations suggest that PKH67 may be used as a specific dye for the labelling and tracing of SMV1 and SSV2. As the viral-host infection experiments were carried out at 78 °C, our study demonstrates the stability of the PKH67 labelling at high temperatures, thus indicating PKH67 as an option for fluorescent labelling used for tracking organisms above physiological temperatures.

### Uptake of SMV1 and SSV2 by human endothelial cells and assessment of cellular safety

**Figure 3:**
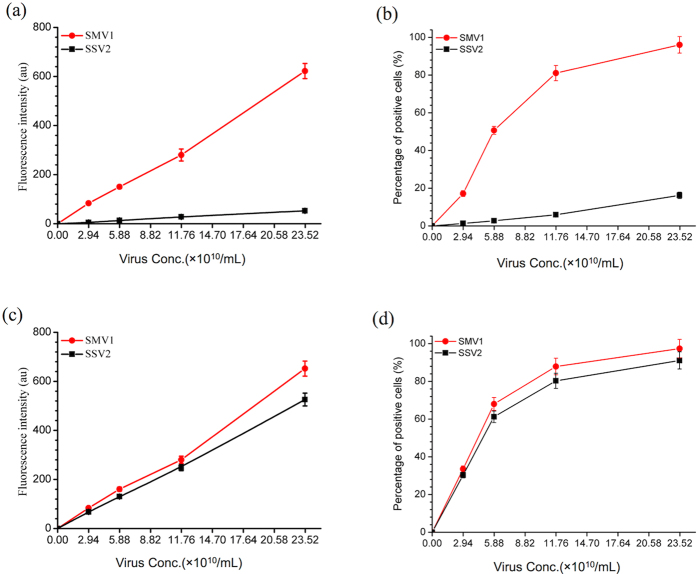
Cellular uptake of PKH67-labelled viruses by hCMEC/D3 (**a,b**) and HUVECs (**c,d**) cells. Left panel: Average fluorescence intensity of cells after exposure to increasing concentration of labeled viruses for 24 h (**a,c**); Right panel: Quantification of positive cells bearing PKH67-labelled viruses by FACS (**b,d**). In all the experiments, corresponding cells were incubated with different concentration of either SMV1-PKH67 or SSV2-PKH67 for 24 h. Viral concentrations were estimated by NTA.

Uptake and intracellular processing of labelled viruses were followed in two phenotypically different human endothelial cell types. The results in Fig. 3 show that HUVECs, but not hCMEC/D3, can interact with both viruses. SMV1 favourably interacts with hCMEC/D3 cells in a concentration-dependent manner, where the proportion of virus-containing cells increases with increasing SMV1 concentration at 24 h post incubation. In contrast to SMV1, hCMEC/D3 cells even at high viral concentrations poorly recognize SSV2. Whether viral adhesion to and recognition by both cell types is mediated through the viral tail regions remains to be elucidated. Nevertheless, the observed differences in viral recognition may be due to different receptor expression and specificity among these cells. Alternatively, these differences may arise from different rates of endocytosis and/or cell-dependent non-specificity in uptake (e.g., through macropinocytosis) processes and require detailed investigation.

**Figure 4:**
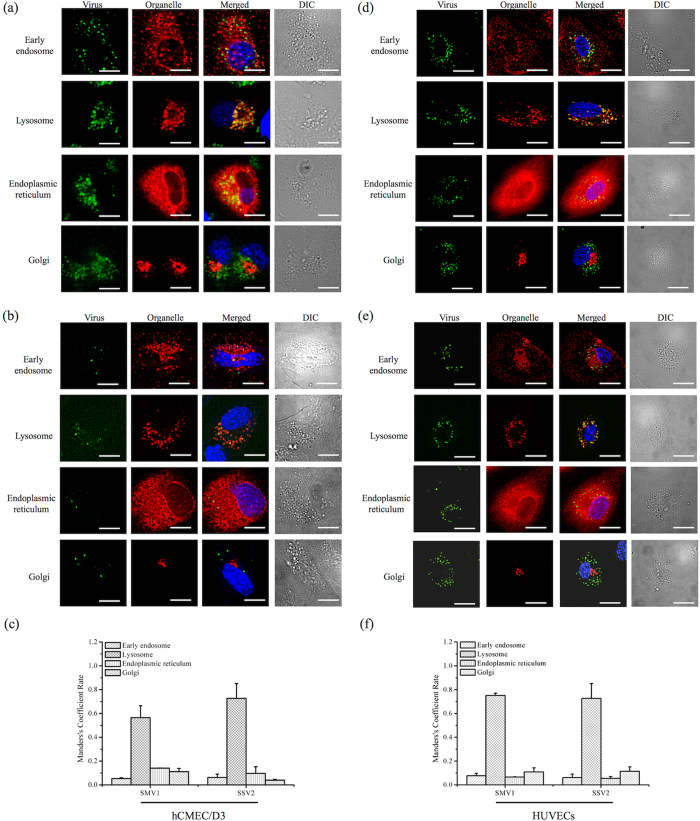
Intracellular trafficking of PKH67-labelled viruses in hCMEC/D3 cells and HUVECs. Intracellular trafficking (live-cell tracking) of nanoparticles in different organelles after 24 h post-treatment with SMV1-PKH67 (**a,d**) and SSV2-PKH67 (**b,e**) viruses. Panels (**a,b**) represent hCMEC/D3 cells and panels (**d,e**) correspond to HUVECs cells. Differential interference contrast (DIC) images were taken simultaneously to show morphological changes and fluorescence positioning. Scale bar = 20 μm. Panels (**c**) and (**f**) represent Manders’ overlap coefficient after image analysis showing the extent of viral-derived fluorescence overlap with the four intracellular compartments in hCMEC/D3 cells and HUVECs, respectively.

Live-cell microscopy confirmed viral internalization in both cell types (Fig. 4). Free dye molecules were not taken up by the cells, when challenged even at higher concentration than the amount of fluorescence in labelled viruses (Supplementary Fig. S4). This confirms that cell associated fluorescence is derived from viral uptake. Tracking studies further revealed predominant localization of viruses to the peri-nuclear region, and particularly to lysosomal compartments, and therefore consistent with endocytic uptake processes^20^,^24^. Due to strong fluorescent signal at the peri-nuclear region, we also investigated viral trafficking to endoplasmic reticulum (ER) and the Golgi complex. In contrast to the lysosomal compartment, Manders’ fluorescence overlap coefficient remains low in both ER and Golgi complex regardless of the virus type. Accordingly, internalization routes such as caveolae-independent and lipid raft/caveolae-mediated endocytosis^25^,^26^, may operate a minor role in viral translocation to these organelles.

**Figure 5:**
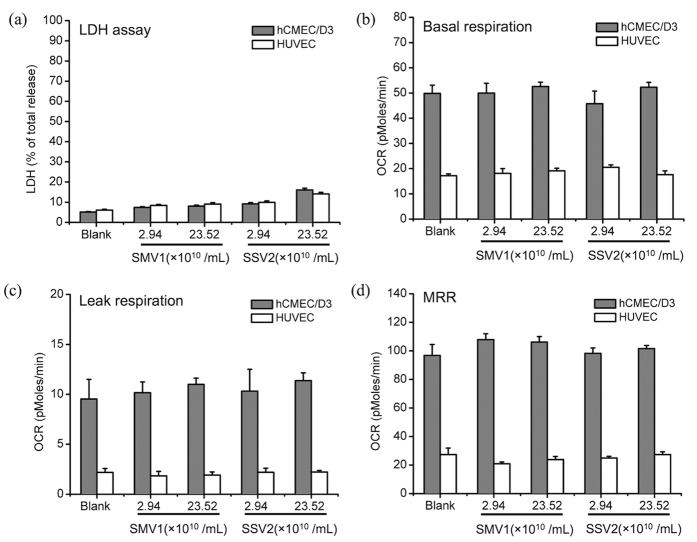
Investigation of cytotoxic effect of viruses in hCMEC/D3 and HUVECs cells. Panel (**a**) shows the extent of lactate dehydrogenase (LDH) release from cells at 24 h post virus treatment. Panels (**b–d**) shows the effect of viral concentration on cellular respiratory states at 24 h post incubation (n = 6). Respiration (indicated as the rate of oxygen consumption) was evaluated at basal state (the physiological coupling state controlled by cellular energy demand) (panel **b**), leak state (where respiration is independent of ADP phosphorylation and mainly occurs due to proton leak from mitochondrial intermembrane space) (panel **c**), and maximum respiratory rate (MRR; signifying the capacity of the mitochondrial electron transport system) (panel **d**). Blank = untreated cells.

Neither SMV1, nor SSV2, induced any deleterious cytotoxic effects. This notion is confirmed from the apparent lack of detrimental effects on cell morphology, LDH release and mitochondrial functionality (Fig. 5) irrespective of virus load, type and the endothelial cell type. Viral challenge also showed no significant changes on basal respiration, mitochondrial proton leak or maximum respiratory rates (MRR) in either cell lines (Fig. 5). This demonstrates that the physiological coupling state of mitochondrial respiration remains unaltered^21^,^22^ even following cellular exposure to high concentrations of either SMV1 or SSV2. This suggestion is further verified by calculations of the coupling efficiency of oxidative phosphorylation (OXPHOS) and the respiratory control ratio (RCR) which revealed no significant changes (Table 1).

### Conclusions and perspective

Our results provide the first insights into the uptake by and behaviour of archaeal viruses in two phenotypically different human endothelial cells *in vitro*. In particular, SSV2 differentiates between HUVECs and hCMEC/D3 cells. The specific recognition and uptake of SMV1 by hCMEC/D3 cells is also interesting and may provide an alternative approach for possible modulation of brain cerebral capillary endothelial cell functions. However, further studies are needed to determine whether viral interaction with mammalian cells is mediated through their tail regions as well as identification of cell surface receptors that participate in viral binding and internalization steps.

As a type of prokaryotes different from bacterial cells, anaerobic archaea have been detected in the human oral, colonic and vaginal microbial flora demonstrating their ability to colonize the human host^11^,^27^,^28^. However, with respect to the safety issue, archaeal viruses have never been reported to integrate into human or any other eukaryotic genomes, and archaeal viral sequences have never been detected in the sequences of eukaryotic genomes. This can be explained by the fact that all known archaeal viral integration mechanisms need the presence of an attachment site and a specific integrase enzyme, which cannot be expressed from the native archaeal viral genome in the eukaryotic cellular environment. Thus, as with plant viruses and bacteriophages^29^, archaeal viruses are less likely to trigger negative downstream effects in mammals due to an inability to proliferate.

In terms of engineering viruses for development as nanovectors, two major techniques are implored. These include genetic engineering and surface functionalization^2^. Although no attempts have been made to genetically modify SSV2 or SMV1, a closely related virus to SSV2 has been shown to be amenable to insertion of genetic material without loss of function^30^. Genome comparison between SMV1 and three related genomes, all retrieved from hot spring metagenomes, revealed that some genes are not present in all. For example, SMV3 has a much larger genome than SMV1, this suggests that large DNA fragments could be inserted into the genome of SMV1 without loss of function^31^. Moreover, several archaeal viruses have been made into genetic model systems, proving the possibility of genetic engineering of archaeal viruses in general^32^. In addition to genetic engineering or by itself, chemoselective chemistry is a powerful tool for incorporating functionalities onto virus scaffolds. Often naturally occurring amino acid residues, suitable for functionalization with an appropriate group, are present on the viral particle surface. The structures of SMV1 and SSV2 have not been resolved, however, the capsid proteins of both viruses contain multiple units of functional groups such as Lys, Asp, and Glu for chemoselective modification, thus making it possible for future surface engineering initiatives.

Another important limitation to using viruses as nanovectors is their physical stability. For many of the contemplated applications, the improvement of the physical stability of viral nanoparticles may be critical to adequately meet the demanding physicochemical conditions they may encounter during production and/or storage^33^. The extremophilic nature of archaeal viruses gives them an inherent stability under a wide range of conditions^7^,^8^. For example, SMV1 demonstrated high structural stability after treatment under a range of harsh conditions including freezing without cryoprotectants^16^. Even more interesting was that SMV1 retained 100% of infectivity after 24 hours storage at −20 °C^16^. Taken together with their unique shape, archaeal viruses may show great promise as future nanoplatforms in bioengineering and development of multifunctional vectors. However, we still need to learn about the interaction between archaeal viruses and the elements of innate immunity to determine their safety^34^.

## Additional Information

**How to cite this article**: Uldahl, K. B. *et al*. Recognition of extremophilic archaeal viruses by eukaryotic cells: a promising nanoplatform from the third domain of life. *Sci. Rep.* **6**, 37966; doi: 10.1038/srep37966 (2016).

**Publisher's note:** Springer Nature remains neutral with regard to jurisdictional claims in published maps and institutional affiliations.

## Supplementary Material

Supplementary Information

## Figures and Tables

**Table 1:** The coupling efficiency of oxidative phosphorylation (OXPHOS) and mitochondrial respiratory control ratio (RCR) in hCMEC/D3 cells and HUVEC cells after 24 h treatment with low and high concentration of different archaeal viruses.

Viruses (×10^10^/mL)	Coupling efficiency OXPHOS		RCR	
	hCMEC/D3	HUVEC	hCMEC/D3	HUVEC
Blank	0.809 ± 0.039	0.874 ± 0.022	10.64 ± 2.06	12.96 ± 2.31
SMV1 (2.94)	0.796 ± 0.015	0.908 ± 0.018	10.79 ± 1.31	12.63 ± 2.47
SMV1 (23.52)	0.791 ± 0.011	0.930 ± 0.047	9.691 ± 0.49	13.10 ± 0.90
SSV2 (2.94)	0.762 ± 0.075	0.893 ± 0.016	10.10 ± 2.05	11.74 ± 1.99
SSV2 (23.52)	0.782 ± 0.014	0.874 ± 0.009	8.99 ± 0.56	12.38 ± 0.75

The coupling efficiency of OXPHOS defines the fraction of protons used for mitochondrial ATP production proportional to protons leaking through the mitochondrial inner membrane. The RCR denotes the capacity of mitochondria to oxidize respiratory substrates and to synthesize ATP. Higher RCR and coupling efficiency of OXPHOS are indicative of mitochondria with high efficiency of substrate oxidation and ATP production.
