# Supplementary material for: Recognition of extremophilic archaeal viruses by eukaryotic cells: a promising nanoplatform from the third domain of life
Source: Sci Rep. 2016 Nov 28;6:37966. doi: 10.1038/srep37966 (PMC5125014; doi:10.1038/srep37966)
Supplement: Supplementary Information [file srep37966-s1.pdf]

Supplementary Information for

Recognition of extremophilic archaeal viruses by eukaryotic cells: a promising nanoplatform from the third domain of life

Kristine Buch Uldahl[1+], Linping Wu[2 +], Arnaldur Hall[2], Pavlos Papathanasiou[1], Xu Peng[1*], Seyed Moein Moghimi[2,3*]

Supplementary Figure S1

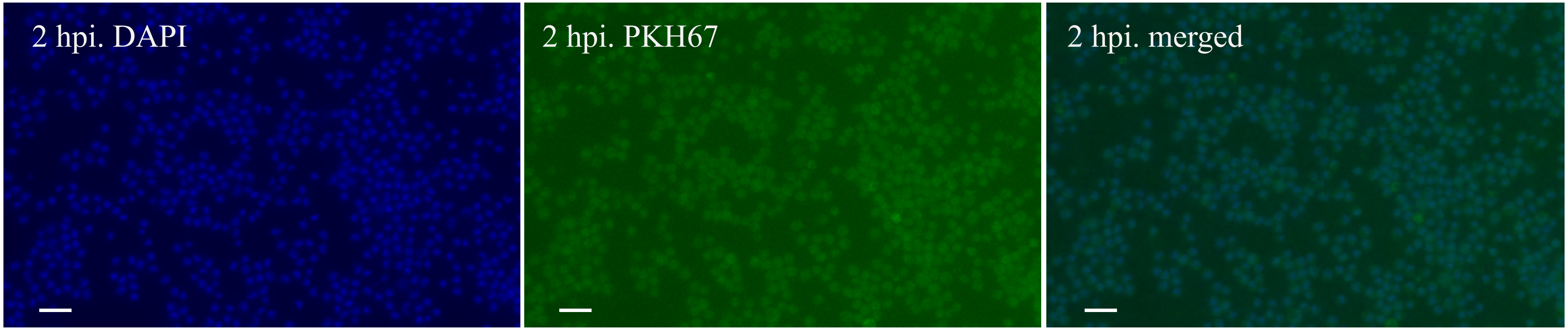

Supplementary Figure S1. **Cell imaging of *S. solfataricus* 5E6 infected with PKH67 labeled SSV2**. Cells were incubated with PKH67 labeled SSV2 and free viral particles were washed away at 2 hours post infection (hpi). The co-localization of DAPI stained cells (blue, left panel) with virus particles (green, middle panel) appears as light purple (right panel). Bar = 9 µm. Cells were imaged with Nikon inverted microscope Eclipse Ti-E (Nikon, Japan) due to the repair and thus the unavailability of the microscope (Zeiss AxioImager Z1 microscope) used for imaging cells presented in Fig. 2 and Fig. S2.

Supplementary Figure S2

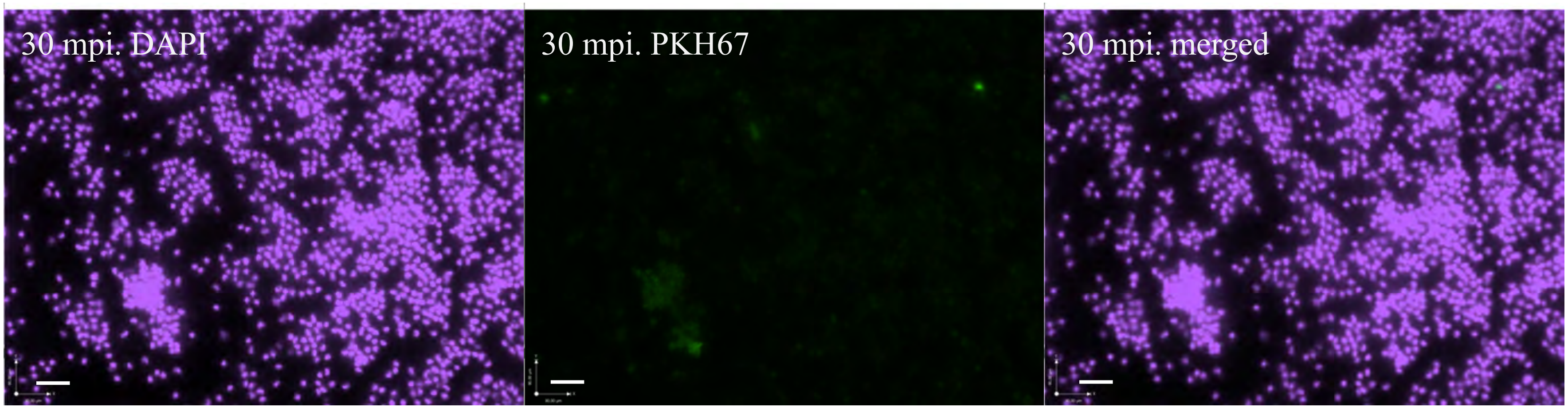

Supplementary Figure S2. **Cell imaging of *S. acidocaldarius* incubated with PKH67 labeled SMV1**. Cells were incubated with PKH67 labeled SMV1 and free viral particles were washed away at 30 min post incubation (mpi). Cells were stained with DAPI (left panel), and no co-localization with DAPI was observed (right panel). Bar = 9 µm. Samples were treated and processed, and images were obtained, in the same way as used in the experiment presented in Fig. 2A.

Supplementary Figure S3

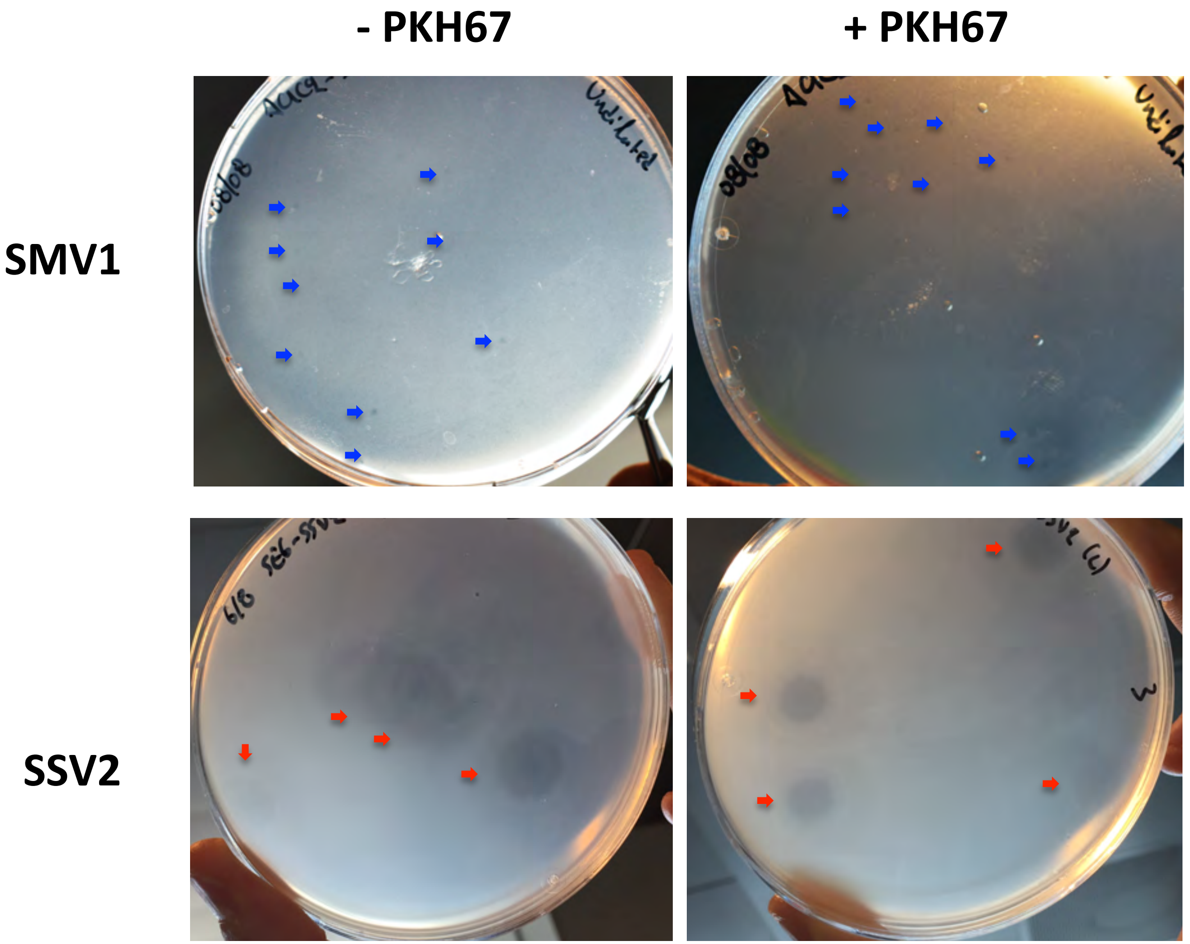

Supplementary Figure S3. **Plaquing efficiency of SMV1 and SSV2 with or without PKH67 labeling**. One preparation of each virus was divided into 2 equal portions and treated with the labeling protocal in the absence (left panel) or presence (right panel) of PKH67 dye. Plaque assays were performed as described in the main text after a 1000x dilution of the treated viruses. SMV1 plaques (indicated with blue arrows) have a diameter of 1-2 mm, some of which appear quite turbid. SSV2 plaques are unusually large, reaching a diameter close to 1 cm (labeled by red arrows). Similar number of plaques derived from labeled and unlabeled viruses indicates that PKH67 has no detrimental effect on plaquing efficiency of the viruses.

Supplementary Figure S4

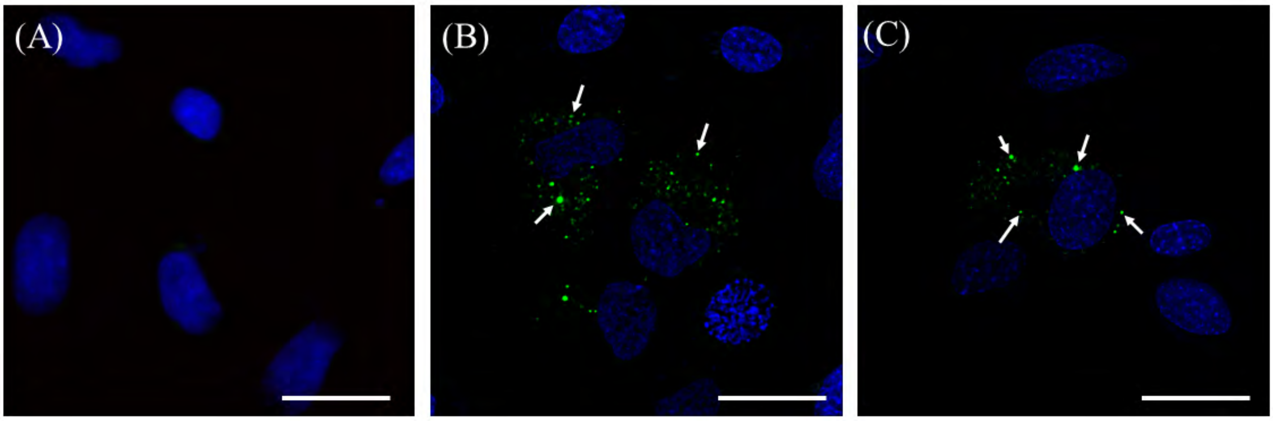

Supplementary Figure S4. Fluorescence microscopy image of PKH67 dye and PKH67-labelled viruses incubated in hCMEC/D3 cells for 24 h. (A) PKH67 dye alone, (B) SMV1-PKH67, (C) SSV2-PKH67. Green: PKH67-labelled viruses, blue: nucleus. Insert bars are 30 µm. Arrows indicate labelled viruses.
